# A single extra copy of Down syndrome critical region 1–4 results in impaired hepatic glucose homeostasis

**DOI:** 10.1016/j.molmet.2018.12.002

**Published:** 2018-12-05

**Authors:** Dong Soo Seo, Gia Cac Chau, Kwan-Hyuck Baek, Sung Hee Um

**Affiliations:** 1 Department of Molecular Cell Biology, Samsung Biomedical Research Institute, Sungkyunkwan University School of Medicine, Suwon, Gyeonggi-do, 16419, South Korea; 2 Department of Health Sciences and Technology, Samsung Advanced Institute for Health Sciences and Technology, Samsung Medical Center, Sungkyunkwan University, Seoul, 06351, South Korea

**Keywords:** Hepatic glucose homeostasis, DSCR1-4, Diabetes, Calcineurin, DSCR1-4, Down syndrome critical region1-4, CsA, cyclosporine A, NFAT, nuclear factor of activated T cells, InsP3Rs, inositol-1,4,5-triphosphate receptors, PTDM, posttransplantation diabetes, GSK3β, glycogen synthase kinase-3 beta, NIK, nuclear factor-κB-inducing kinase

## Abstract

**Objectives:**

During fasting, hepatic gluconeogenesis is induced to maintain energy homeostasis. Moreover, abnormal dysregulation of hepatic glucose production is commonly observed in type 2 diabetes. However, the signaling components controlling hepatic glucose production to maintain normal glucose levels are not fully understood. Here, we examined the physiological role of Down syndrome critical region 1–4 (DSCR1-4), an endogenous calcineurin signaling inhibitor in the liver that mediates metabolic adaptation to fasting.

**Methods:**

We assessed the effect of cyclosporine A, an inhibitor of calcineurin signaling on gluconeogenic gene expression in primary hepatocytes. DSCR1-4 expression was examined in diet- and genetically-induced mouse models of obesity. We also investigated the metabolic phenotype of a single extra copy of *DSCR1-4* in transgenic mice and how DSCR1-4 regulates glucose homeostasis in the liver.

**Results:**

Treatment with cyclosporin A increased hepatic glucose production and gluconeogenic gene expression. The expression of DSCR1-4 was induced by refeeding and overexpressed in obese mouse livers. Moreover, transgenic mice with a single extra copy of *DSCR1-4* exhibited pyruvate intolerance and impaired glucose homeostasis. Mechanistically, DSCR1-4 overexpression increased phosphorylation of the cAMP response element-binding protein, which led to elevated expression levels of gluconeogenic genes and, thus, enhanced hepatic glucose production during fasting.

**Conclusion:**

A single extra copy of DSCR1-4 results in dysregulated hepatic glucose homeostasis and pyruvate intolerance. Our findings suggest that nutrient-sensitive DSCR1-4 is a novel target for controlling hepatic gluconeogenesis in diabetes.

## Highlights

• DSCR1 mRNA and protein levels are increased in livers upon nutrient availability.
• DSCR1-4 is overexpressed in diet- or genetically induced obesity.
• DSCR1-4 trisomy mice exhibit impaired glucose homeostasis and pyruvate intolerance.
• Trisomy of DSCR1-4 leads to increased hepatic glucose production.

## 1 Introduction

Glucose homeostasis is tightly controlled to meet the energy requirements of mammals [1]. Under fasting conditions, glucagon secreted by pancreatic α-cells induces hepatic gluconeogenesis to maintain nutrient levels [2]. Gluconeogenesis is regulated by transcriptional activation of gluconeogenesis rate-limiting enzymes, such as phosphoenolpyruvate carboxykinase (PEPCK) and glucose 6-phosphatase (G6Pase) [1]. In response to low levels of glucose, protein kinase A becomes activated and mediates phosphorylation of cAMP response element-binding protein (CREB) regions, which leads to increased binding of CREB to the cAMP-responsive element (CRE) in the promoter of gluconeogenic genes [3]. CREB requires the binding of its coactivator CREB-regulated transcription coactivator2 (CRTC2) to increase the transcriptional activity of gluconeogenic genes [4]. Additionally, activated calcineurin dephosphorylates and thereby promotes nuclear translocation of CRTC2, which leads to the induction of gluconeogenic gene expression [5]. This suggests a role for the calcineurin-CREB-CRTC2 axis in regulation of glucose metabolism.

Calcineurin is a calcium/calmodulin-activated serine/threonine phosphatase that consists of the catalytic A and regulatory B subunits [6] and is the target of certain immunosuppressants, such as cyclosporine A (CsA) and tacrolimus [7]. Recent studies demonstrated that calcineurin signaling is involved in glucose homeostasis [5], [8]. Overexpression of calcineurin in the liver increases hepatic glucose production [5]. By contrast, knockdown of calcineurin in the liver results in reduced expression of gluconeogenic genes [5]. Calcineurin dephosphorylates nuclear factor of activated T cells (NFAT), leading to its activation and thereby enhancing the activity of the insulin gene promoter upon glucose treatment [9]. Additionally, NFATc2- and NFATc4-deficient mice exhibit increased insulin sensitivity and are protected against high fat diet-induced obesity [10]. These studies suggest that calcineurin signaling contributes to glucose and insulin homeostasis. However, how the regulators of calcineurin signaling affect energy homeostasis during fasting is not fully understood.

The calcineurin signaling regulator inositol-1,4,5-triphosphate receptors (InsP3Rs) increases calcineurin activity in response to glucagon, whereas inhibition of InsP3Rs in response to insulin blocks calcineurin signaling [5]. RNA interference-mediated InsP3Rs or calcineurin knockdown in mice improves glucose tolerance [5]. Another regulator of calcineurin signaling is Down syndrome critical region 1-4 (DSCR1-4, also known as MCIP1, Calcipressin1, RCAN1, and Adapt78), which physically interacts with calcineurin and inhibits its phosphatase activity [11]. Although several studies have demonstrated the effects of regulators in calcineurin signaling on the control of glucose metabolism, the role of DSCR1-4 in glucose homeostasis has not been clearly defined. All DSCR1 isoforms possess seven exons, which contain a calcineurin-binding motif [11] and can be alternatively spliced to produce three isoforms, such as 1-1L, 1-1S, and 1-4 in humans [11]. Interestingly, the DSCR1 gene is overexpressed in those with Down syndrome [12].

The prevalence of diabetes in children with Down syndrome is three-fold higher than in normal children [13], [14], [15]. Additionally, metabolic syndrome and type 2 diabetes occur at relatively early ages in those with Down syndrome [16]. Although an increased incidence of diabetes in patients with Down syndrome has been reported for many years, the molecular basis of dysregulated glucose homeostasis in patients with Down syndrome is not well understood. In this study, we investigated the role of DSCR1-4 in the liver, which mediates metabolic adaptation in response to fasting, by introducing a single extra copy of DSCR1-4 into mice.

## 2 Materials and methods

### 2.1 Mice

Transgenic mice with a single extra copy of *DSCR1-4* (trisomy) were obtained from Dr. Sandra Ryeom (University of Pennsylvania, USA) [12]. Eight-week-old male *ob/ob* mice were purchased from Jackson Laboratory (Bar Harbor, ME, USA). All mice were backcrossed every ten generations onto the C57BL/6J background and maintained on a 12-h light/dark cycle. C57BL/6J high fat diet (HFD)-fed mice (60% kcal%, Research Diets, Inc., New Brunswick, NJ, USA; D12492) were obtained from Jung Ang Laboratory Animal (Korea). All animal experiments were performed in accordance with the guidelines of the Institutional Animal Care and Use Committee of Sungkyunkwan University School of Medicine, which is an Association for Assessment and Accreditation of Laboratory Animal Care International accredited facility that abides by the institute of Laboratory Animal Resources guide.

### 2.2 Animal experiments

Eight-to 10-week-old male mice were fed a normal chow diet *ad libitum*. To prepare the diet-induced obesity model, 6-week-old male mice were fed the HFD for 8 weeks. Blood glucose levels were measured in the tail vein using a glucometer (Roche, Basel, Switzerland). Mice were fasted for 16 h and then the pyruvate tolerance test was performed by measuring the plasma glucose levels after intraperitoneal (i.p.) injection of sodium pyruvate (2 g/kg body weight; Sigma, St. Louis, MO, USA). For the insulin tolerance test, mice were fasted for 4 h and then injected i.p. with insulin (0.75 Ug/kg body weight; GIBCO, Grand Island, NY, USA).

### 2.3 Isolation of primary hepatocytes and cell culture

Primary hepatocytes were isolated from 8- to 10-week-old mice using collagenase-based methods as previously described [17]. Primary hepatocytes were seeded onto 6-well-plates and maintained in Medium 199 (M199, Sigma) supplemented with 10% fetal bovine serum (GIBCO), 100 U/mL penicillin, 100 μg/mL streptomycin, 23 mM HEPES, and 10 nM dexamethasone (Sigma).

### 2.4 Plasmid and transfection

HEK293T cells were maintained with Dulbecco's modified Eagle's medium (Welgene, Daegu, Korea) supplemented with 10% fetal bovine serum, 100 U/mL penicillin, and 100 μg/mL streptomycin. HEK293T cells were transfected with a plasmid expressing *myc*-tagged *DSCR1-4* using Lipofectamine (Invitrogen, Carlsbad, CA, USA).

### 2.5 Glucose production assay

Glucose production was evaluated in Krebs-ringer buffer containing 20 mM sodium lactate, 2 mM sodium pyruvate, and 100 nM dexamethasone (Sigma) after 8 h. The glucose levels in the Krebs-ringer buffer were measured using a QuantiChrom™ glucose assay kit (Bioassay Systems, Hayward, CA, USA) and normalized to protein concentrations.

### 2.6 *In vivo* imaging

Adenovirus expressing *G6Pase* (−231/+57) luciferase (Ad-*G6Pase-Luc*) was kindly provided by Dr. SH. Koo (Korea University, KOREA). Ad-*G6Pase-Luc* (1 × 10^8^ pfu/kg of body weight) was injected into the tail veins of WT and *DSCR1-4* transgenic mice. At 3 days after injection, mice were fasted for 6 h and then *in vivo* luciferase activity was measured after luciferin injection using an IVIS lumina imaging system (Caliper Life Sciences, Hopkinton, MA, USA).

### 2.7 Immunoblotting and immunoprecipitation

Immunoblotting and immunoprecipitation were performed as described previously [17], [18]. Antibodies against DSCR1 were purchased from Sigma–Aldrich. Antibodies against p-AKT (S473), p-CREB (S133), p-eIF2α (S51), eIF2α, XBP1s, AKT, HSP90 were purchased from Cell Signaling Technology (Danvers, MA, USA). Antibodies against CREB, Myc, α-tubulin, and β-actin were purchased from Santa Cruz Biotechnology, Inc. (Dallas, TX, USA). The antibody against TORC2 and Calcineurin B were purchased from Merck (Billerica, MA, USA). The bands were detected using an enhanced chemiluminescence system (Pierce, Rockford, IL, USA).

### 2.8 RNA isolation, reverse transcriptase (RT)-PCR, and quantitative real-time (qRT)-PCR

RNA isolation, RT-PCR, and qRT-PCR were performed as described previously [17]. The primer sets were 5′-GGTCTGGACTTCTCTGCCAAG-3′ and 5′-CTGTCTTGCTTTCGATCCTGG-3′ for *PEPCK*; 5′-GTGAATTACCAAGACTCCCAGG-3′ and 5′-CCATGGCATGGCCAGAGG-3′ for *G6Pase*; 5′-AGCTCCCTGATTGCTTGTGT-3′ and 5′-AGGAACTCGGTCTTGTGCAG-3′ for *DSCR1-4*; 5′-GAGGATTTTGCTAACCTGACACC-3′ and 5′-TTGACGGTAACTGACTCCAGC-3′ for *ATF3*; 5′-GGTCTGCTGAGTCCGCAGCAGG-3′ and 5′-AGGCTTGGTGTATACATGG-3′ for *XBP1s*; 5′-CCACCACACCTGAAAGCAGAA-3′ and 5′-AGGTGA AAGGCAGGGACTCA-3′ for *CHOP*; 5′-ACTTGGGGACCACCTATTCCT-3′ and 5′-ATCGCCAATCAGACGCTCC -3′ for *BIP*; 5′-GTGCGGAGAAGGTTCAAGG-3′ and 5′-CTTAGAGGACACATTGTGAGCAA-3′ for *L32*.

### 2.9 Statistical analysis

Data are presented as mean ± standard error of the mean (SEM). The main and interactive effects were analyzed by analysis of variance or Student's *t*-test. Differences between individual group means were analyzed by post-hoc Bonferroni test or unpaired two-tailed *t-*test. A *p* value of <0.05 was considered statistically significant.

## 3 Results

### 3.1 Cyclosporin A increases gluconeogenesis in hepatocytes

**Figure 1:**
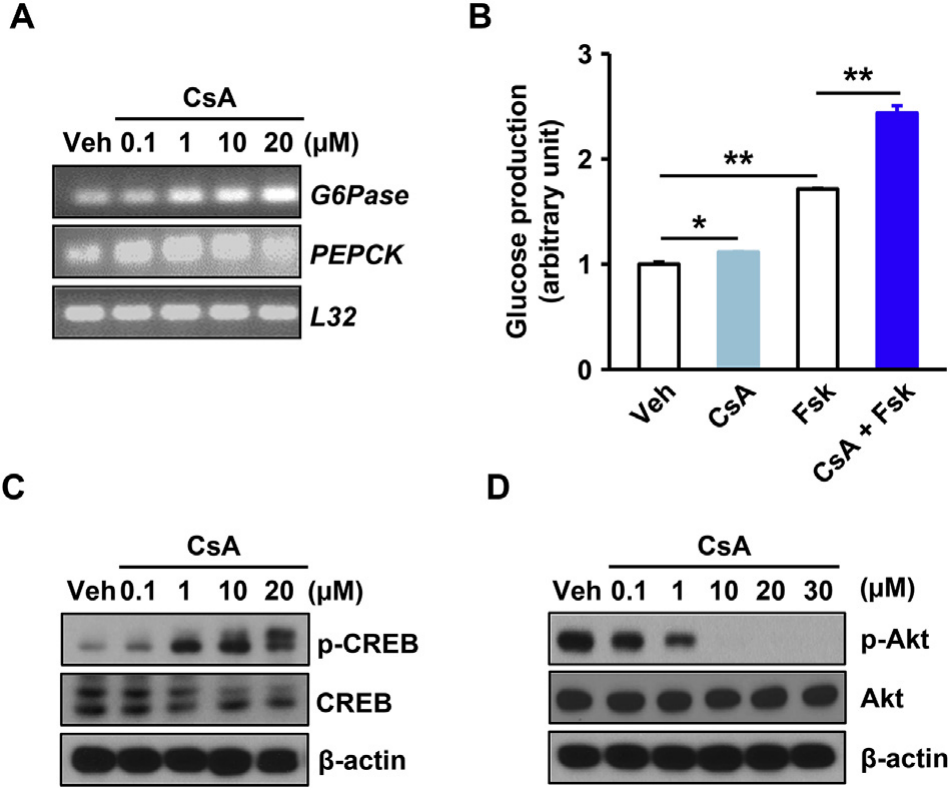
**Cyclosporin A induces gluconeogenesis in primary mouse hepatocytes.** (**A**) Levels of *G6Pase* and *PEPCK* mRNA in primary hepatocytes after treatment with cyclosporine A (CsA) at the indicated concentration for 24 h. (**B**) Glucose production assays were performed in hepatocytes treated with vehicle, Forskolin (Fsk, 10 μM), or CsA (10 μM) for 8 h (n = 3 per group). (**C**) Immunoblots showing phosphorylation of CREB in primary hepatocytes after treatment with CsA at the indicated concentration. (**D**) Immunoblots showing the phosphorylation levels of Akt in primary hepatocytes after treatment with CsA at the indicated concentration. Assays and blots are representative of three independent experiments. *p < 0.05, **p < 0.01; *t*-test, Values indicate the mean ± SEM.

CsA forms a complex with cytosolic cyclophilin A, which competitively binds to and inhibits the activity of calcineurin [19]. CsA is an immunosuppressant used for organ transplantation, an inhibitor of calcineurin signaling, and is associated with post-transplant diabetes mellitus (PTDM) [20]. However, it remains unclear how inhibition of calcineurin signaling is linked to PTDM in the liver. To determine whether CsA affects hepatic glucose metabolism, we treated hepatocytes with CsA and examined the expression of gluconeogenic genes. The mRNA levels of gluconeogenic enzyme, such as *G6Pase* and *PEPCK,* were increased in wild-type (WT) hepatocytes upon treatment with CsA (Figure 1A). Moreover, treatment with Forskolin, an adenylate cyclase agonist that elevates cyclic AMP levels, further enhanced CsA-induced glucose production (Figure 1B).

Upon fasting, phosphorylated CREB directly activates the transcription of gluconeogenic genes [1]. We next examined phosphorylation of CREB in hepatocytes upon treatment with CsA. CsA treatment of WT hepatocytes led to increased phosphorylation of CREB in a dose-dependent manner (Figure 1C and Supplementary Fig. 1A), suggesting that CsA induces phosphorylation of CREB, thus leading to increased expression of gluconeogenic genes. Considering that phosphorylation of Akt promotes nuclear exclusion of FoxO1 that blocks the transcriptional activity of gluconeogenic genes [21], we further examined the phosphorylation levels of Akt in primary hepatocytes after treatment with CsA. The phosphorylation of Akt was reduced in a dose-dependent manner by treatment with CsA (Figure 1D and Supplementary Fig. 1B). Together, these results suggest that CsA, an inhibitor of calcineurin signaling, increases hepatic glucose production, which may be relevant in the development of PTDM.

### 3.2 DSCR1-4 expression is induced in response to nutrients and overexpressed in obese mouse livers

**Figure 2:**
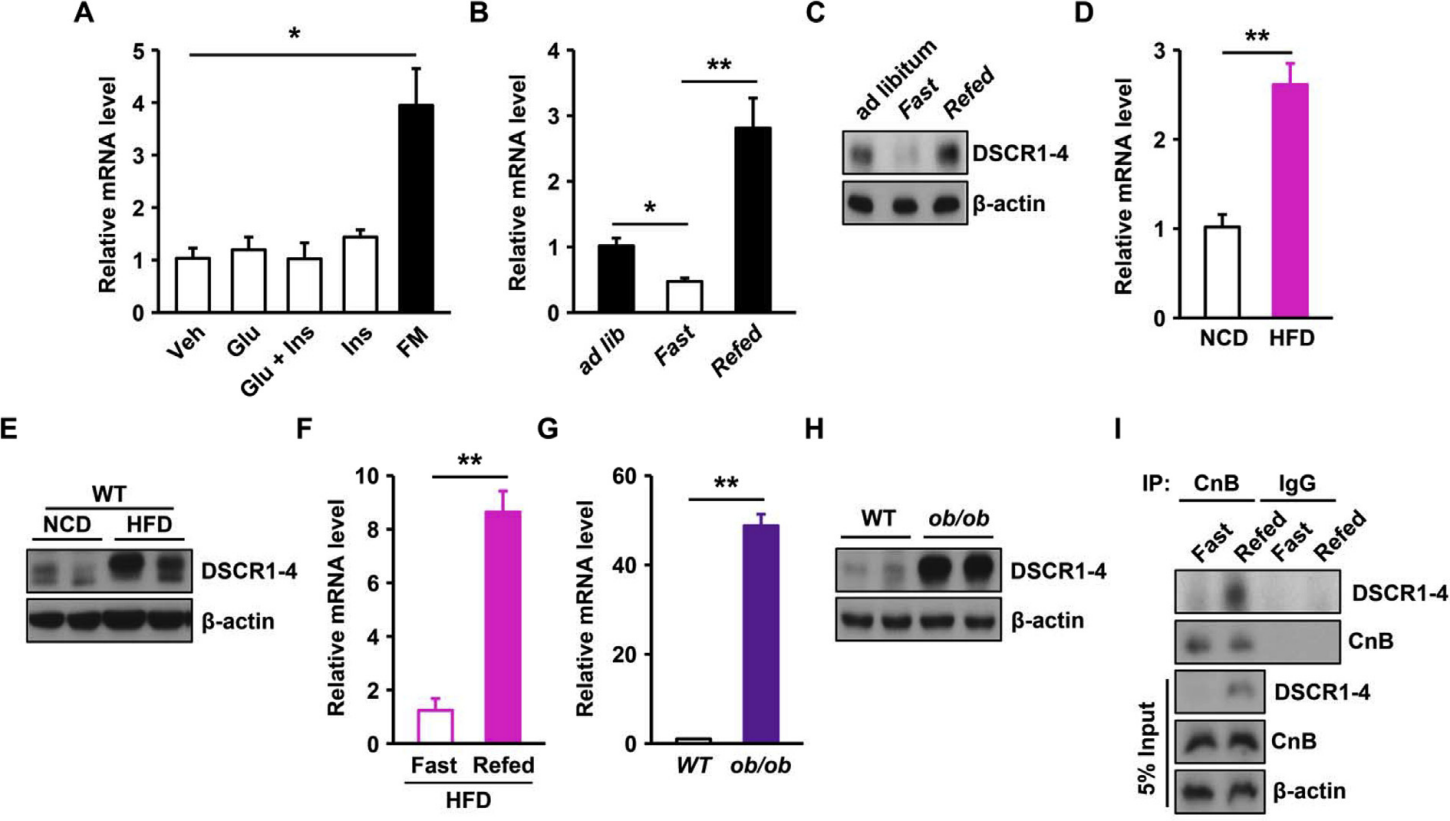
**Expression of DSCR1-4 is induced by refeeding and overexpressed in diet-induced obesity.** (**A**) Levels of *DSCR1-4* mRNA in hepatocytes after treatment with glucose (25 mM), insulin (10 nM), or complete DMEM (FM, full media) in primary hepatocytes from WT mice (n = 3 per group). (**B, C**) DSCR1-4 (**B**) mRNA and (**C**) protein levels 4 in livers from WT mice after feeding *ad libitum* and then fasting for 18 h or followed by refeeding for 1 h (n = 3 per group). (**D, E**) DSCR1-4 (**D**) mRNA and (**E**) protein levels in livers from mice fed with normal chow diet or high-fat diet after 24 h fasting (n = 3 per group). (**F**) Levels of *DSCR1-4* mRNA in livers of high fat diet-fed mice after 24 h fasting or followed by refeeding for 1 h (n = 3 per group). (**G, H**) DSCR1-4 (**G**) mRNA and (**H**) protein levels in livers from WT and *ob/ob* mice (n = 3 per genotype). (**I**) Liver extracts from WT mice after fasting or followed by refeeding for 1 h were subjected to immunoprecipitation using anti-Calcineurin B (CnB) antibodies and then analyzed by western blotting with the indicated antibodies. Assays and blots are representative of three independent experiments. *p < 0.05, **p < 0.01; *t*-test, values indicate the mean ± SEM.

Considering that treatment with CsA increased the expression of genes related to gluconeogenesis and subsequent enhanced glucose production (Figure 1), we next examined whether DSCR1, a negative regulator of calcineurin signaling, is also related to hepatic glucose metabolism. To investigate the metabolic function of DSCR1-4 in the liver, we first determined the expression levels of DSCR1-4 in hepatocytes under different culture conditions. The expression of DSCR1-4 was increased in full media compared to in HBSS media (Figure 2A), whereas either glucose or insulin alone or a combination of both did not affect the levels of DSCR1-4, suggesting that expression of DSCR1-4 is mainly induced by the nutrients present in full media.

To determine whether expression of DSCR1-4 changes in livers in response to nutrient levels, we measured the expression of DSCR1-4 in mouse livers after feeding *ad libitum*, fasting, or refeeding. The mRNA and protein levels of DSCR1-4 were decreased in livers of fasted mice, but increased in livers of refed mice or mice fed *ad libitum* (Figure 2B,C and Supplementary Fig. 1C), indicating that DSCR1-4 expression has a positive correlation with nutrient availability. To determine whether DSCR1-4 expression is altered in a pathological state, such as obesity, we measured the expression levels of DSCR1-4 in the livers of diet- or genetically-induced mouse models of obesity. The *DSCR1-4* mRNA levels were increased by approximately three-fold and protein levels were dramatically increased in the livers of mice fed a HFD compared to those fed a normal chow diet (Figure 2D,E and Supplementary Fig. 1D). Consistently, upon HFD feeding, the hepatic mRNA levels of *DSCR1-4* were significantly increased in refed mice compared to fasted mice (Figure 2F), suggesting that DSCR1-4 expression in the liver is related to nutritional status and is markedly increased in diet-induced obesity. Moreover, the mRNA and protein levels of DSCR1-4 were dramatically increased in the livers of *ob/ob* mice compared to those of WT mice (Figure 2G,H and Supplementary Fig. 1E). Given that DSCR1-4 inhibits the phosphatase activity of calcineurin by directly associating with this protein [11], we next determined whether the interaction between DSCR1-4 and calcineurin is affected in livers after refeeding. Immunoprecipitation revealed the association between DSCR1-4 and calcineurin was stronger in livers of refed mice than in fasted mice (Figure 2I and Supplementary Fig. 1F), indicating DSCR1-4 forms more complexes with calcineurin upon nutrient enrichment. These results suggest that the expression levels of DSCR1-4 are linked to nutrient-sensitive metabolism in the liver and DSCR1-4 is aberrantly overexpressed in obesity.

### 3.3 *DSCR1-4* transgenic mice exhibit impaired glucose homeostasis and pyruvate intolerance

**Figure 3:**
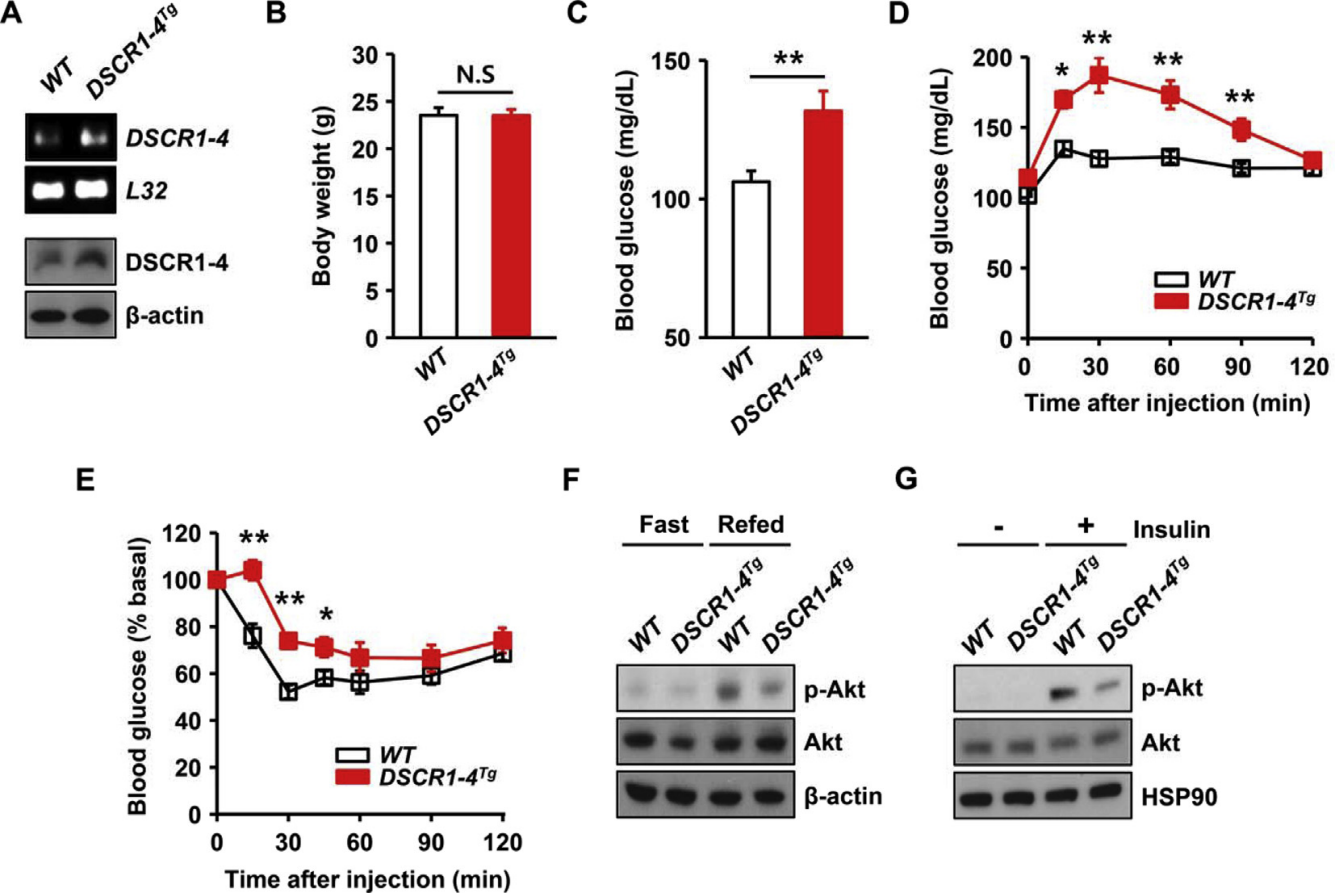
**Transgenic mice with a single extra copy of *DSCR1-4* exhibit impaired glucose homeostasis and pyruvate intolerance**. (**A**) DSCR1-4 mRNA and protein levels in WT and *DSCR1-4* transgenic livers. (**B**) Body weights were measured in 10-week-old WT and *DSCR1-4* transgenic mice (n = 6 per genotype). (**C**) Blood glucose levels were monitored in 10-week- old WT and *DSCR1-4* transgenic mice after fasting for 24 h (n = 4 per genotype). (**D**) Pyruvate tolerance tests were performed for WT and *DSCR1-4* transgenic mice after 16 h of fasting (n = 5 per genotype). (**E**) Insulin tolerance tests were performed for WT and *DSCR1-4* transgenic mice after 4 h of fasting (n = 5 per genotype). (**F**) Immunoblots showing the phosphorylation levels of Akt in livers from WT mice after fasting for 24 h or followed by refeeding for 1 h. (**G**) Immunoblots showing the levels of Akt phosphorylation in *WT* and *DSCR1-4* transgenic hepatocytes after treatment with insulin (100 nM) for 30 min. Assays and blots are representative of three independent experiments. *p < 0.05, **p < 0.01; ANOVA, values indicate the mean ± SEM.

Based on the data showing that DSCR1-4 expression is sensitive to nutrient levels (Figure 2), we investigated the effect of DSCR1-4 on glucose homeostasis *in vivo.* First, we measured the body weight of transgenic mice (Figure 3A). Transgenic mice with a single extra copy of *DSCR1-4* had normal body weights (Figure 3B). Considering our data showing treatment with CsA increases the expression of gluconeogenic genes (Figure 1), we next determined whether DSCR1-4 affects glucose levels after fasting. *DSCR1-4* transgenic mice exhibited significantly higher blood glucose levels than WT mice after fasting for 24 h (Figure 3C), indicating that DSCR1-4 plays a critical role in the maintenance of glucose homeostasis during fasting. In response to fasting, plasma glucose is generated and maintained by hepatic gluconeogenesis, which produces glucose from pyruvate [1]. To determine whether a single extra copy of DSCR1-4 influences glucose production in the liver, we next performed a pyruvate tolerance test to measure the systemic elevation of glucose derived from pyruvate. After administration of pyruvate, *DSCR1-4* transgenic mice displayed dramatically increased blood glucose levels compared to WT mice (Figure 3D), suggesting that overexpression of DSCR1-4 increases glucose production in the liver using pyruvate. Considering that pyruvate tolerance affects insulin sensitivity [22] and insulin-induced activation of Akt suppresses hepatic gluconeogenesis upon feeding [21], we next performed an insulin tolerance test. *DSCR1-4* transgenic mice were insulin resistant compared to WT mice (Figure 3E). Consistent with this, phosphorylation levels of Akt were decreased in *DSCR1-4* transgenic livers compared to WT livers after refeeding (Figure 3F and Supplementary Fig. 1G). We further examined the phosphorylation levels of Akt in primary hepatocytes after treatment with insulin. The phosphorylation levels of Akt were decreased in *DSCR1-4* transgenic hepatocytes compared to WT hepatocytes upon insulin treatment (Figure 3G and Supplementary Fig. 1H), suggesting that mice with a single extra copy of *DSCR1-4* have decreased insulin-mediated Akt phosphorylation, which leads to enhanced hepatic gluconeogenesis.

Endoplasmic reticulum (ER) stress is induced by feeding [23] and is related to insulin resistance [24]. We next determined whether ER stress-mediated signaling was upregulated in *DSCR1-4* transgenic livers. The expression of ER stress genes, such as *activating transcription factor 3 (ATF3), X-box binding protein 1 transcription factor (XBP1s), CCAAT-enhancer binding protein homologous protein (CHOP),* and *binding immunoglobulin protein (BIP)* was not altered in *DSCR1-4* transgenic livers after refeeding (Supplementary Fig. 2A). In addition, the protein levels of ER stress-signaling components, such as phosphorylated eukaryotic translation initiation factor 2A (eIF2α), were similar between WT and *DSCR1-4* transgenic livers upon refeeding (Supplementary Fig. 2B). These results indicate that ER stress is unlikely to be linked to nutrient-induced DSCR1-4 overexpression.

Together, these data suggest DSCR1-4 negatively regulates hepatic glucose homeostasis and induces pyruvate intolerance in an ER stress-independent manner.

### 3.4 Trisomy of DSCR1-4 leads to increased hepatic glucose production

**Figure 4:**
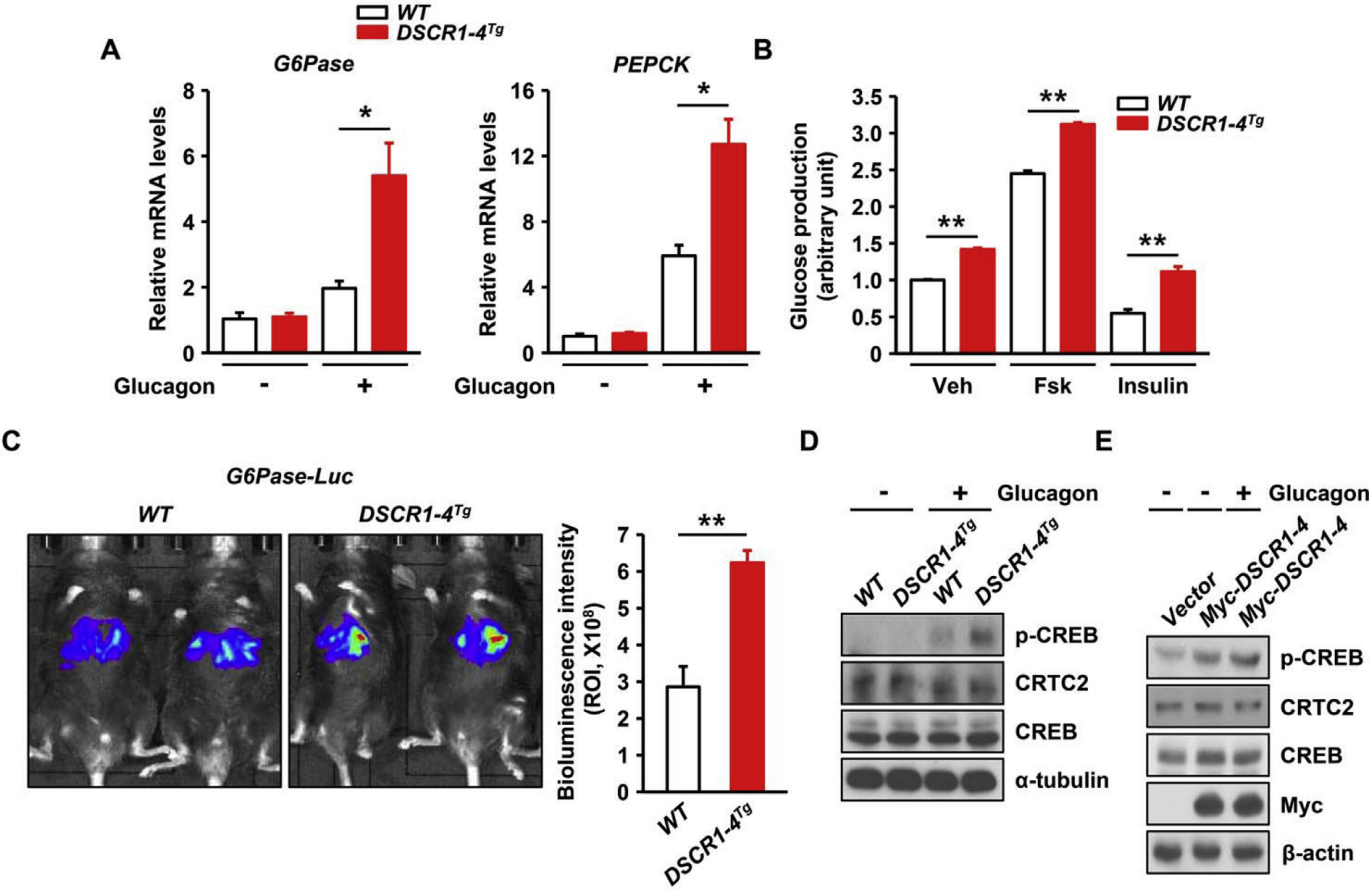
**Trisomy of DSCR1-4 results in increased hepatic glucose production.** (**A**) *G6Pase* or *PEPCK* mRNA levels in WT or *DSCR1-4* transgenic hepatocytes after treatment with glucagon (100 nM) for 2 h (n = 3 per group). (**B**) Glucose production in WT or *DSCR1-4* transgenic hepatocytes after treatment with Forskolin (Fsk) (10 μM) or insulin (100 nM) (n = 3 per group). (**C**) Hepatic *G6Pase*-luciferase (Ad-*WT G6Pase* [−231/+57]-*Luc*) activities in livers of WT or *DSCR1-4* transgenic mice after 6 h fasting (n = 3 per genotype). (**D**) Immunoblots showing the phosphorylation levels of CREB and CRTC2 in WT or *DSCR1-4* transgenic hepatocytes after treatment with glucagon (100 nM). (E) Immunoblotting showing the phosphorylation levels of CREB and CRTC2 in cells overexpressing DSCR1-4 after glucagon treatment (100 nM). Assays and blots are representative of three independent experiments. *p < 0.05, **p < 0.01; *t*-test, values indicate the mean ± SEM.

During an insulin-resistant state, insulin cannot suppress hepatic glucose production, which results in abnormal activation of the glucagon-mediated gluconeogenesis program [25]. Based on this, we next assessed gluconeogenesis in primary hepatocytes from *DSCR1-4* transgenic mice. Upon glucagon treatment, the expression levels of gluconeogenic genes were markedly elevated in *DSCR1-4* transgenic hepatocytes compared to those in WT hepatocytes (Figure 4A), suggesting that DSCR1-4 increases gluconeogenesis in hepatocytes by enhancing the expression of gluconeogenic genes. Furthermore, *DSCR1-4* transgenic hepatocytes displayed increased glucose production upon treatment with Forskolin or insulin compared to WT mice (Figure 4B). To determine whether the transcriptional activity of gluconeogenic genes is physiologically increased by a single extra copy of DSCR1-4 in mice, we performed luciferase bioluminescence imaging to measure the *in vivo* transcriptional activity of gluconeogenic genes. This system is highly sensitive because it is not influenced by auto-fluorescence or the excitation light source [26]. Transgenic mice with a single extra copy of *DSCR1-4* exhibited markedly increased *G6Pase* transcriptional activity *in vivo* under fasting conditions compared with WT mice (Figure 4C). These data suggest that DSCR1-4 regulates hepatic glucose homeostasis and gluconeogenic gene expression.

During fasting, glucagon stimulates protein kinase A-CREB signaling and induces the dephosphorylation of CRTC2 [4]. We next examined the phosphorylation levels of CREB and CRTC2 upon stimulation with glucagon. CREB phosphorylation was markedly increased in *DSCR1-4* transgenic hepatocytes after glucagon treatment compared to WT hepatocytes (Figure 4D and Supplementary Fig. 1I). However, there was no difference in the dephosphorylation levels of CRTC2 between *DSCR1-4* transgenic and WT hepatocytes (Figure 4D and Supplementary Fig. 1I). Similarly, overexpression of DSCR1-4 resulted in increased phosphorylation of CREB in HEK293 cells, whereas dephosphorylation levels of CRTC2 were unaltered (Figure 4E and Supplementary Fig. 1J), indicating DSCR1-4-induced gluconeogenesis is related to increased phosphorylation of CREB, but not CRTC2. Collectively, these results suggest that overexpression of DSCR1-4 increases the phosphorylation of CREB, leading to the induction of hepatic gluconeogenesis.

## 4 Discussion

During fasting, increasing levels of circulating glucagon maintain glucose homeostasis by stimulating gluconeogenesis [2]. In type 2 diabetes, abnormally increased hepatic glucose production is accompanied by constitutive activation of the gluconeogenic program [27]. However, regulators of signaling pathways that control the levels of blood glucose, a main energy source during fasting, have not been fully elucidated. Here, we show that a single extra copy of DSCR1-4 in mice impaired hepatic glucose homeostasis. Consistent with our findings, overexpression of DSCR1-1 resulted in glucose intolerance and hypoinsulinemia along with β-cell mitochondrial dysfunction [28], [29]. Thus, these findings and our own suggest that DSCR1 is a novel physiological regulator that controls metabolic adjustment to fasting by increasing hepatic gluconeogenesis and, thus, contributing to whole-body energy homeostasis.

Our analysis further revealed that DSCR1-4 was aberrantly overexpressed in the livers of mice with dietary- or genetically induced obesity. Intriguingly, expression of *DSCR1* is also increased in islets of human diabetes and after chronic high glucose treatment [28]. Our results and those of other studies suggest that the expression of DSCR1-4 is sensitive to the nutritional status. Calcineurin enhances the transcriptional activity of NFAT [6] and is downregulated by refeeding [5] and our data show that DSCR1-4 is strongly associated with calcineurin in response to nutrient enrichment. Therefore, we speculate that the expression of DSCR1-4 increases its association with calcineurin, leading to the inhibition of its activity possibly through negative feedback of calcineurin-NFAT-DSCR1-4 signaling. Future studies should reveal such networks by determining whether DSCR1-4 inhibits NFAT transcriptional activity through calcineurin in the liver and whether DSCR1-4 affects calcineurin-induced nuclear translocation of NFAT in response to refeeding.

ER stress is induced in the feeding state [23] and is related to the development of insulin resistance [24]. Upon ER stress, c-Jun upregulates DSCR1 expression, resulting in suppression of ER-stress responses in mouse embryo fibroblasts [30]. However, in our analysis, there was no apparent difference between the expression of ER stress-related genes and proteins in WT and *DSCR1-4* transgenic mouse livers. Thus, it is unlikely that insulin resistance in *DSCR1-4* transgenic mice originates from ER stress.

During feeding, insulin-mediated activation of PI3K/Akt signaling results in the inhibition of hepatic gluconeogenesis [25]. Consistent with insulin resistance and pyruvate intolerance in *DSCR1-4* transgenic mice, levels of Akt phosphorylation were also decreased in hepatocytes from these mice. DSCR1-4 overexpression-mediated decreases in Akt phosphorylation may be related to hyperglycemia and pyruvate intolerance through increased hepatic glucose production. At present, it remains unclear how DSCR1-4 regulates Akt signaling during maintenance of hepatic glucose homeostasis. Inhibition of calcineurin decreases Akt phosphorylation in INS-1 cells [31] and our data shows reduced Akt phosphorylation in *DSCR1-4* transgenic mice upon refeeding. Therefore, it is possible that DSCR1-4 signaling regulates Akt phosphorylation through calcineurin. It would be of interest to determine whether DSCR1-4 directly regulates Akt phosphorylation in response to nutritional status and whether DSCR1-4 affects the activity of upstream effectors of Akt, such as insulin receptor substrate, phosphatidylinositol-4,5-bisphosphate 3-kinase, and protein 3-phosphoinositide-dependent protein kinase-1, in the liver.

Our results demonstrated that CsA increased expression of gluconeogenic genes in hepatocytes. Consistent with this, a previous study has reported that certain immunosuppressants used to prevent organ rejection, such as CsA and sirolimus, contribute to the development of PTDM [20]. Considering that DSCR1 binds to calcineurin and suppresses its phosphatase activity, overexpression of DSCR1 is thought to inactivate calcineurin [11]. RNA interference-mediated calcineurin knockdown in mice leads to reduced expression of gluconeogenic genes and decreased circulating blood glucose, while adenoviral overexpression of calcineurin in mice has the opposite effects [5]. Similarly, ablation of calcineurin globally or in skeletal muscle protects against obesity [32]. By contrast, our analysis revealed transgenic mice with a single extra copy of *DSCR1-4* exhibited enhanced gluconeogenic gene expression, along with increased levels of hepatic glucose production during fasting. Unexpectedly, *DSCR1-4* transgenic hepatocytes had levels of CRTC2 similar to those of WT hepatocytes, whereas phosphorylation of CREB was elevated upon glucagon treatment. Previous studies have shown CRTC2-deficient mice have normal glucose levels during both feeding and fasting conditions without any effects on hepatic glucose production [33], suggesting the contribution of CRTC2 to glucose homeostasis is limited. Additionally, serum increases the proliferation of vascular smooth muscle cells by inducing phosphorylation of CREB without regulating CRTC2 [34]. Thus, our results suggest DSCR1-4 induces gluconeogenesis by increasing the phosphorylation of CREB independently of CRTC2 phosphorylation.

It remains unclear how DSCR1-4 enhances CREB phosphorylation to lead to increased hepatic glucose production. It has been reported that phosphorylation of CREB is regulated by glycogen synthase kinase-3 beta (GSK3β) [35], orphan nuclear receptor small heterodimer partner [36], and mitogen-activated protein kinase p38 [37]. Interestingly, DSCR1 increases the expression of GSK3β in PC-12 cells [38] and nuclear factor-κB-inducing kinase (NIK) induces hyperglycemia by phosphorylating CREB in obesity [39] and DSCR1 stability [40]. Based on these findings, DSCR1-4 may promote hepatic gluconeogenesis by regulating GSK3β or NIK, resulting in increased phosphorylation of CREB. Given that phosphorylated CREB binds to the promoter of gluconeogenic genes to enhance their transcriptional activity [1], how DSCR1-4 mechanistically regulates hepatic glucose homeostasis should be further evaluated.

Notably, elevated glucose production in the liver is one of the major determinants of increased fasting plasma glucose levels in patients with type 2 diabetes [27], [41]. Our analysis revealed that a single extra copy of DSCR1-4 increases hepatic glucose production and expression of gluconeogenic genes, resulting in pathological states, such as insulin resistance and pyruvate intolerance. These phenotypes partially recapitulate the dysregulated metabolic homeostasis occurring in those with Down syndrome. Thus, our findings suggest DSCR1-4 is a novel potential endogenous target for impaired glucose homeostasis in patients with type 2 diabetes and Down syndrome.

## Author contributions

DS and GC performed all experiments and collected the data. KB reviewed the manuscript and contributed to the discussion. DS and SU designed the study, analyzed data, and wrote the manuscript. All authors have read and approved the final version of the manuscript.
