# Supplementary material for: A single extra copy of Down syndrome critical region 1–4 results in impaired hepatic glucose homeostasis
Source: Mol Metab. 2018 Dec 5;21:82–9. doi: 10.1016/j.molmet.2018.12.002 (PMC6407364; doi:10.1016/j.molmet.2018.12.002)
Supplement: Multimedia component 1 [file mmc1.pdf]

Supplementary Figure 1

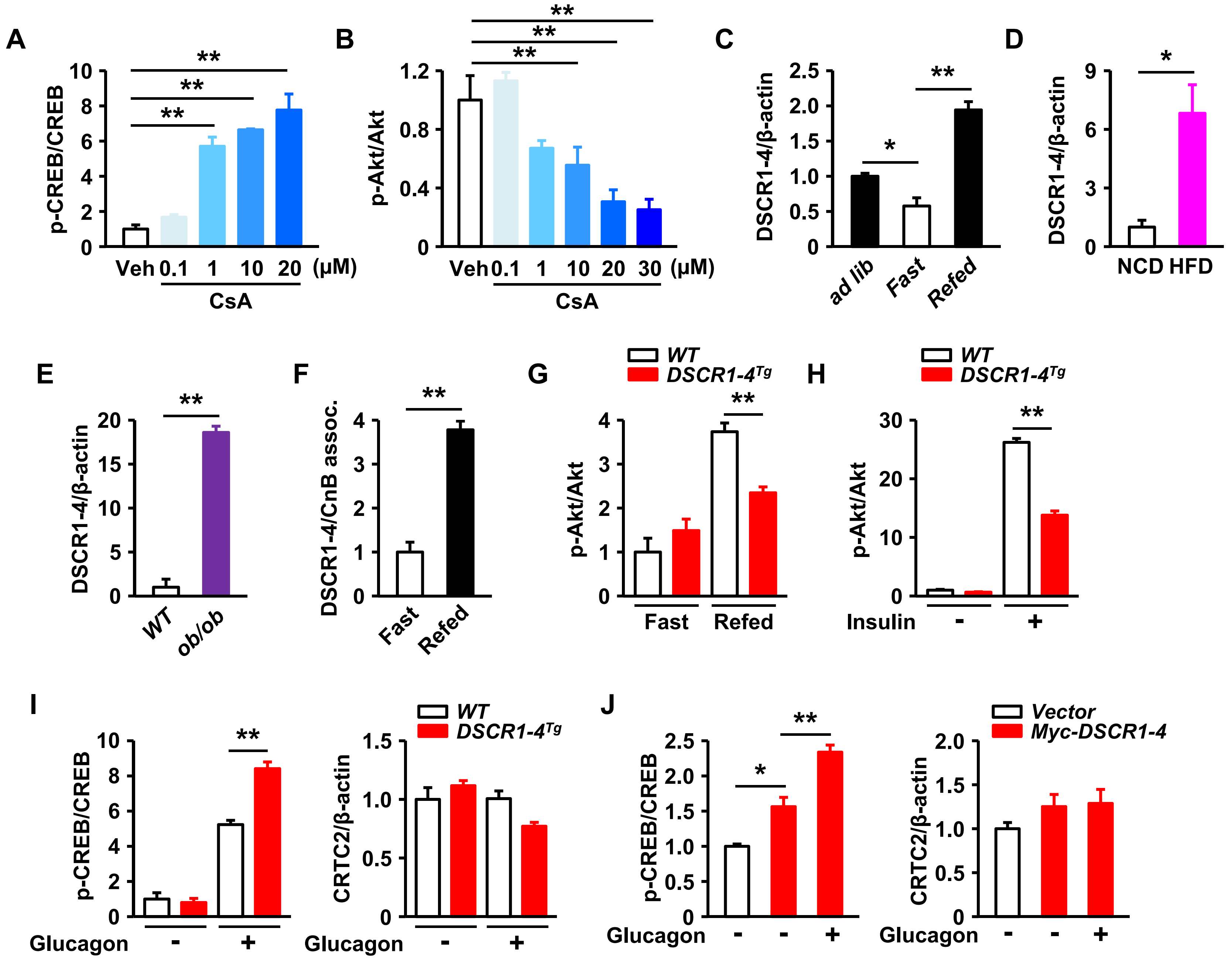

Supplementary Figure 1. Quantification of western blot data

Quantification of western blots data from three independent experiments was performed using Image J software. Quantification analysis of (**A**) the relative levels of p-CREB/total CREB in Figure 1C. (**B**) The relative levels of p-Akt/total Akt in Figure 1D. (**C-E**) The relative levels of DSCR1-4/β-actin in Figure 2C, 2E, and 2H. (**F**) The relative association levels of DSCR1-4/CnB in Figure 2I. (**G, H**) The relative levels of p-Akt/total Akt in Figure 3F and 3G. (**I, J**) The relative levels of p-CREB/total CREB in Figure 4D and 4E. $^{*}p < 0.05$, $^{**}p < 0.01$; t-test, values indicate the mean ± SEM.

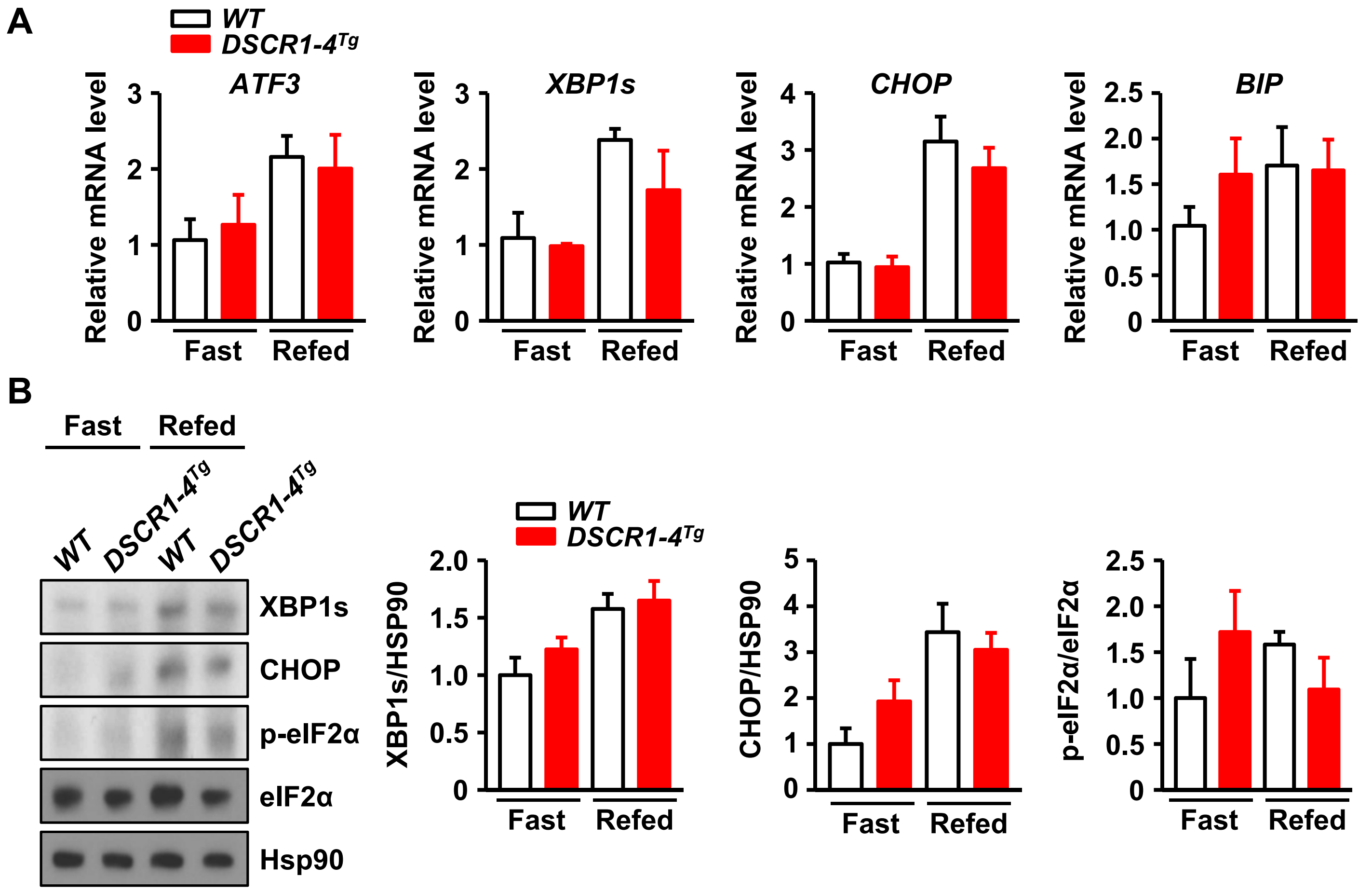

Supplementary Figure 2. A single extra copy of *DSCR1-4* does not affect mRNA or protein levels of ER stress-related genes and signaling

(**A**) mRNA levels of ER stress-related gene in livers from WT mice after fasting for 24 h or followed by refeeding for 1 h. (**B**) Immunoblots showing ER stress-related signaling proteins in livers from WT mice after fasting for 24 h or followed by refeeding for 1 h. Quantification of the relative levels of XBP1s/HSP90, CHOP/HSP90, and p-eIF2α/total eIF2α. Assays and blots are representative of three independent experiments. *$p < 0.05$, ** $p < 0.01$; t-test, values indicate the mean ± SEM.
